# Supplementary material for: HIVprotI: an integrated web based platform for prediction and design of HIV proteins inhibitors
Source: J Cheminform. 2018 Mar 9;10:12. doi: 10.1186/s13321-018-0266-y (PMC5845081; doi:10.1186/s13321-018-0266-y)
Supplement: Supplementary file 2 — Additional file 2. Source code of HIVProtI web server. [file 13321_2018_266_MOESM2_ESM.zip › HIVprotI_Source-code/hivprotI/weka-3-6-6/doc/weka/core/converters/AbstractSaver.html]

AbstractSaver


---


|  |  |  |
| --- | --- | --- |
| **Overview** \| **Package** \| **Class** \| **Tree** \| **Deprecated** \| **Index** \| **Help** \| **Weka's home** | |  |
| **PREV CLASS**   **NEXT CLASS** | **FRAMES**    **NO FRAMES**     **All Classes** |  |
| SUMMARY: NESTED \| FIELD \| CONSTR \| METHOD | DETAIL: FIELD \| CONSTR \| METHOD |  |


---


## weka.core.converters Class AbstractSaver

```
java.lang.Object
  weka.core.converters.AbstractSaver
```

**All Implemented Interfaces:**: java.io.Serializable, CapabilitiesHandler, Saver, RevisionHandler

**Direct Known Subclasses:**: AbstractFileSaver, DatabaseSaver

---

``` public abstract class AbstractSaver extends java.lang.Object implements Saver, CapabilitiesHandler ```

Abstract class for Saver

**Version:**
:   $Revision: 1.4 $

**Author:**
:   Richard Kirkby (rkirkby@cs.waikato.ac.nz), Stefan Mutter (mutter@cs.waikato.ac.nz)

**See Also:**: Serialized Form

---

**Field Summary**

**Fields inherited from interface weka.core.converters.Saver**

`BATCH, INCREMENTAL, NONE`


**Constructor Summary**

`AbstractSaver()`


| **Method Summary** | |
| --- | --- |
| `void` | `cancel()`             Cancels the incremental saving process if the write mode is CANCEL. |
| `java.lang.String` | `filePrefix()`             Default implementation throws an IOException. |
| `Capabilities` | `getCapabilities()`             Returns the Capabilities of this saver. |
| `java.lang.String` | `getFileExtension()`             Default implementation throws an IOException. |
| `Instances` | `getInstances()`             Gets instances that should be stored. |
| `int` | `getWriteMode()`             Gets the write mode. |
| `void` | `resetOptions()`             resets the options |
| `void` | `resetStructure()`             Resets the structure (header information of the instances) |
| `java.lang.String` | `retrieveDir()`             Default implementation throws an IOException. |
| `void` | `setDestination(java.io.File file)`             Default implementation throws an IOException. |
| `void` | `setDestination(java.io.OutputStream output)`             Default implementation throws an IOException. |
| `void` | `setDir(java.lang.String dir)`             Default implementation throws an IOException. |
| `void` | `setDirAndPrefix(java.lang.String relationName, java.lang.String add)`             Default implementation throws an IOException. |
| `void` | `setFile(java.io.File file)`             Default implementation throws an IOException. |
| `void` | `setFilePrefix(java.lang.String prefix)`             Default implementation throws an IOException. |
| `void` | `setInstances(Instances instances)`             Sets instances that should be stored. |
| `void` | `setRetrieval(int mode)`             Sets the retrieval mode. |
| `int` | `setStructure(Instances headerInfo)`             Sets the strcuture of the instances for the first step of incremental saving. |
| `abstract  void` | `writeBatch()`             Writes to a file in batch mode To be overridden. |
| `void` | `writeIncremental(Instance i)`             Method for incremental saving. |

**Methods inherited from class java.lang.Object**

`equals, getClass, hashCode, notify, notifyAll, toString, wait, wait, wait`

**Methods inherited from interface weka.core.RevisionHandler**

`getRevision`

**Constructor Detail**

### AbstractSaver

```
public AbstractSaver()
```


**Method Detail**

### resetOptions

```
public void resetOptions()
```

:   resets the options

---


### resetStructure

```
public void resetStructure()
```

:   Resets the structure (header information of the instances)

---


### setRetrieval

```
public void setRetrieval(int mode)
```

:   Sets the retrieval mode.

    :   **Specified by:**: `setRetrieval` in interface `Saver`
    :   **Parameters:**: `mode` - the retrieval mode

---


### getWriteMode

```
public int getWriteMode()
```

:   Gets the write mode.

    :   **Specified by:**: `getWriteMode` in interface `Saver`
    :   **Returns:**: the write mode

---


### setInstances

```
public void setInstances(Instances instances)
```

:   Sets instances that should be stored.

    :   **Specified by:**: `setInstances` in interface `Saver`
    :   **Parameters:**: `instances` - the instances

---


### getInstances

```
public Instances getInstances()
```

:   Gets instances that should be stored.

    :   **Returns:**: the instances

---


### setDestination

```
public void setDestination(java.io.File file)
                    throws java.io.IOException
```

:   Default implementation throws an IOException.

    :   **Specified by:**: `setDestination` in interface `Saver`
    :   **Parameters:**: `file` - the File **Throws:**: `java.io.IOException` - always

---


### setDestination

```
public void setDestination(java.io.OutputStream output)
                    throws java.io.IOException
```

:   Default implementation throws an IOException.

    :   **Specified by:**: `setDestination` in interface `Saver`
    :   **Parameters:**: `output` - the OutputStream **Throws:**: `java.io.IOException` - always

---


### getCapabilities

```
public Capabilities getCapabilities()
```

:   Returns the Capabilities of this saver. Derived savers have to
    override this method to enable capabilities.

    :   **Specified by:**: `getCapabilities` in interface `CapabilitiesHandler`
    :   **Returns:**: the capabilities of this object **See Also:**: `Capabilities`

---


### setStructure

```
public int setStructure(Instances headerInfo)
```

:   Sets the strcuture of the instances for the first step of incremental saving.
    The instances only need to have a header.

    :   **Parameters:**: `headerInfo` - an instances object. **Returns:**: the appropriate write mode

---


### cancel

```
public void cancel()
```

:   Cancels the incremental saving process if the write mode is CANCEL.

---


### writeIncremental

```
public void writeIncremental(Instance i)
                      throws java.io.IOException
```

:   Method for incremental saving.
    Standard behaviour: no incremental saving is possible, therefore throw an
    IOException.
    An incremental saving process is stopped by calling this method with null.

    :   **Specified by:**: `writeIncremental` in interface `Saver`
    :   **Parameters:**: `i` - the instance to be saved **Throws:**: `java.io.IOException` - IOEXception if the instance acnnot be written to the specified destination

---


### writeBatch

```
public abstract void writeBatch()
                         throws java.io.IOException
```

:   Writes to a file in batch mode
    To be overridden.

    :   **Specified by:**: `writeBatch` in interface `Saver`
    :   **Throws:**: `java.io.IOException` - exception if writting is not possible

---


### getFileExtension

```
public java.lang.String getFileExtension()
                                  throws java.lang.Exception
```

:   Default implementation throws an IOException.

    :   **Specified by:**: `getFileExtension` in interface `Saver`
    :   **Returns:**: a string conatining the file extension (including the '.') **Throws:**: `java.io.IOException` - always: `java.lang.Exception` - exception if a Saver not implementing FileSourcedConverter is used.

---


### setFile

```
public void setFile(java.io.File file)
             throws java.io.IOException
```

:   Default implementation throws an IOException.

    :   **Specified by:**: `setFile` in interface `Saver`
    :   **Parameters:**: `file` - the File **Throws:**: `java.io.IOException` - always

---


### setFilePrefix

```
public void setFilePrefix(java.lang.String prefix)
                   throws java.lang.Exception
```

:   Default implementation throws an IOException.

    :   **Specified by:**: `setFilePrefix` in interface `Saver`
    :   **Parameters:**: `prefix` - the file prefix **Throws:**: `java.io.IOException` - always: `java.lang.Exception` - exception if a Saver not implementing FileSourcedConverter is used.

---


### filePrefix

```
public java.lang.String filePrefix()
                            throws java.lang.Exception
```

:   Default implementation throws an IOException.

    :   **Specified by:**: `filePrefix` in interface `Saver`
    :   **Returns:**: the prefix of the file name **Throws:**: `java.io.IOException` - always: `java.lang.Exception` - exception if a Saver not implementing FileSourcedConverter is used.

---


### setDir

```
public void setDir(java.lang.String dir)
            throws java.io.IOException
```

:   Default implementation throws an IOException.

    :   **Specified by:**: `setDir` in interface `Saver`
    :   **Parameters:**: `dir` - the name of the directory to save in **Throws:**: `java.io.IOException` - always

---


### setDirAndPrefix

```
public void setDirAndPrefix(java.lang.String relationName,
                            java.lang.String add)
                     throws java.io.IOException
```

:   Default implementation throws an IOException.

    :   **Specified by:**: `setDirAndPrefix` in interface `Saver`
    :   **Parameters:**: `relationName` -: `add` - **Throws:**: `java.io.IOException` - always

---


### retrieveDir

```
public java.lang.String retrieveDir()
                             throws java.io.IOException
```

:   Default implementation throws an IOException.

    :   **Specified by:**: `retrieveDir` in interface `Saver`
    :   **Returns:**: the directory as a string **Throws:**: `java.io.IOException` - always


---


|  |  |  |
| --- | --- | --- |
| **Overview** \| **Package** \| **Class** \| **Tree** \| **Deprecated** \| **Index** \| **Help** \| **Weka's home** | |  |
| **PREV CLASS**   **NEXT CLASS** | **FRAMES**    **NO FRAMES**     **All Classes** |  |
| SUMMARY: NESTED \| FIELD \| CONSTR \| METHOD | DETAIL: FIELD \| CONSTR \| METHOD |  |


---
